# Study of parameters in focus simulation functions of virtual slide

**DOI:** 10.1186/1746-1596-6-S1-S24

**Published:** 2011-03-30

**Authors:** Ichiro Mori, Takashi Ozaki, Emiko Taniguchi, Kennichi Kakudo

**Affiliations:** 1 Department of Human Pathology, Wakayama Medical University 811-1,Kimiidera,Wakayama City,Wakayama 641-8509, Japan

## Abstract

As a special function of Virtual Slide (VS) for thick specimens like cytology slides, multilayer (Z-stack) simulated focus and focus fusion were introduced. From the standpoint of surgical pathologist, the optimum parameters for multilayer focus simulation were examined. First, minimal thickness of the layer was checked by measuring thickness of small cells counting the number of the layers that come into focus. Then the optimal number of layers to scan, total thickness, was tried. Small-sized cell nuclei showed around 2μm or less thickness. As minimal thickness of one layer for focus simulation, less than 2 μm is required. Papillary cell mass of urothelial carcinoma, aspiration cytology specimen of breast or thyroid, and uterine cervical smear showed different optimal thickness. Cells piling up more than 4 to 5 layer are difficult to make close up observation. Total 15 (to 30) μm thick scan was enough for most specimens. The “focus fusion” image is single layer image synthesized from multiple layer images. Several layer thicknesses were examined, and there was negligible difference between the focus fusion image synthesized from 0.25 and 1μm thick layers. In the focus fusion image synthesized from 3μm thick layers, some cells not to come into focus. The “focus fusion” seems to contain all the cells in one plane, and easy for screening. To emphasize the existence of myoepithelial cells in fibroadenoma of breast, or to clarify the 3-dimensional tissue structure, multilayer image was better. From our results, 10 layers with 1.5μm thick each provide sufficient information in most specimens.

## Introduction

Virtual slide technology is now introduced to many fields[1-5]. Among these, cytology slide diagnosis is remaining as a big challenge because it requires focus control and high resolution[6]. Focus simulation using multilayer or Z-stack and focus fusion were introduced in late years. Our aim is to decide the optimal parameters for focus simulation functions, the thickness of each layer and how many layers should we cut.

## Material and methods

**Figure 1:**
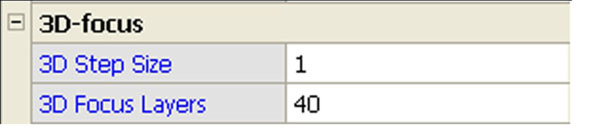
Focus simulation control menu. “3D Step Size” sets layer thickness with value “1” for 0.25μm. “3D Focus Layers” sets the total number of layers with value “40” for 40 layers on both side of autofocus plane. This setting, 3D Step Size: 1 and 3D Focus Layers: 40, results 0.25 X (40+1+40), almost 20μm as total thickness to scan.

The virtual slide scanner we used is TOCO from Claro, the tiling type scanner that has multilayer and focus fusion function (Figure 1). The nuclear width was measured using scale bar on the screen provided by virtual slide viewer. The thickness of the nuclei was measured by counting the layers that come into focus. The viewer has button to change focus planes with one click for one layer. At least ten cells were count.

To decide the optimum layer thickness for multilayer focus simulation, we should know whether the cells in cytology slide keeps 3-dimensional shape or deformed by preparation procedure. We tried to check the 3-D shape of lymphocyte nuclei using formalin-fixed, paraffin section because the paraffin section is expected to keep original nuclear shape. We made 10μm thick paraffin section of spleen from an autopsy case, and then scanned with layers of 0.25μm thick each. We carefully select lymphocytes that has whole nucleus in the 10μm section, then count the number of layers that come into focus.

Next, we checked the 3-D shape of lymphocyte nuclei in thick cytology specimen. Lymphocytes in breast aspiration cytology specimen were measured. We also checked lymphocytes in Giemsa slide that uses drying fixation and improperly dried lymphocytes in Papanicolaou slides.

Then the 3-D shape of various cells was measured. We measured nuclei of myoepithelial cells and duct epithelial cells from the breast fibroadenoma specimen together with the distance between these cells. Small-sized cells like surface squamous epithelial cells and shrunk cells in urine were also measured. Based on these data, optimal layer thickness is considered.

To decide the total thickness to scan, thick specimens like papillary cell mass of urothelial carcinoma, aspiration cytology specimen of breast or thyroid were selected, and tried to decide how many stacked nuclei are possible to diagnose.

In “focus fusion” image, the same layer thickness is possible to indicate. Several layer thicknesses were examined.

## Results

The lymphocyte nuclei in the paraffin section of spleen have 4.3 to 4.5μm diameter, and 16 to 20 layers thickness, that correspond approximately to 4 to 5μm thickness. Lymphocyte nuclei in the thick breast aspiration cytology specimen have diameter of about 4.7 to 5.4μm, and 16 to 20 layers that correspond to 4 to 5μm. In the months-old slides, the lymphocyte nuclei showed similar diameter, but the thickness decreased to about 2 to 3μm. We checked Giemsa slide that uses drying fixation and improperly dried cells in Papanicolaou slides. The lymphocyte nuclei were flattened to 1.25 to 2.5μm (Table 1).

**Table 1:** Nuclear size of lymphocyte

	Diameter	Thickness
Spleen (Formalin-fixed paraffin section)	4.3 - 4.5	4 - 5
Breast (aspiration cytology)	4.7 - 5.4	4 - 5
Giemsa (pleural effusion)	5.1 - 6.4	1.25 - 1.5

Next, we measured myoepithelial cells from the breast fibroadenoma. The myoepithelial cell nuclei had rugby ball-like shape with longer axis for 5.9 to 7.5μm and shorter axis for 2.2 to 3.2μm. Nuclear thickness was about 2.0 to 2.5μm. The duct epithelial cell nuclei of the same breast fibroadenoma showed fried egg-like shape with 6.8 to 8.5μm diameter and 2.0 to 3.0μm thickness (Table 2).

**Table 2:** Nuclear size of various cells

	Diameter (long)	Diameter (short)	Thickness
Myoepithelial cell nuclei	5.9 - 7.5	2.2 - 3.2	2.0 - 2.5
Duct epithelial cell nuclei	6.8 - 8.5	6.8 - 8.5	2.0 - 3.0
Surface squamous epithelial cell	4.4 - 4.7	4.4 - 4.7	1 - 1.25
Middle squamous epithelial cells	8.5 - 10.1	6.4 - 7.2	2.0 - 2.5
Urothelial cells	5.4 - 8.5	4.4 - 5.4	2.0 - 4.25

**Figure 2:**
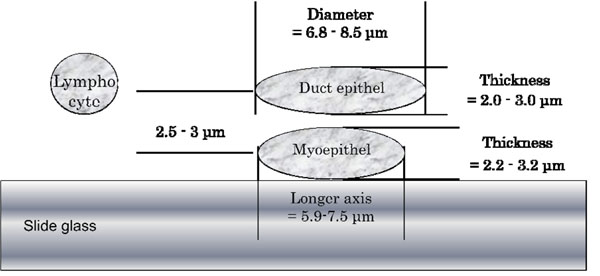
Schematic side view of breast duct cells on slide glass in scaled size

The depth direction distance between myoepithelial cells and duct epithelial cells was measured counting the layers between the myoepithelial cell nuclei’s largest diameter to the duct epithelial cell nuclei’s largest diameter, which was about 2.5 to 3μm. Figure 2 shows schematic side view of these breast duct cells in scaled size.

As far we tried, more than 5 to 6 nuclei stacked together was difficult to distinguish, and not good for diagnosis use. Total thickness to scan also depends on the size of the nuclei. The largest nucleus we found in this study was urothelial carcinoma cell in urine that has 50μm diameter and about 10μm thickness.

Using the “focus fusion”, several layer thicknesses were examined, and there was negligible difference between the focus fusion image synthesized from 0.25 and 1μm thick layers. In the focus fusion image synthesized from 3μm thick layers, some cells occasionally disappears or not to come into focus.

## Discussion

Using scale bar on the screen for diameter, and count the layers that come into focus for the thickness, the lymphocyte nuclei in formalin-fixed, paraffin section showed almost spherical shape. This result assures that we can use our virtual slide in semi-morphometrical analysis. Then we measured lymphocyte nuclei in thick cytology slide, and found that they also keep spherical shape still after embedding in the medium. Nuclear size was about 4 to 5μm, smaller than the value shown in histology textbook (6 to 15μm)[7]. This result is possibly due to preparation procedure. Histological slide suffered formalin fixation and heat coagulation in melted paraffin, and cytological slide suffers alcoholic fixation. In the months-old slides, the nuclei diameter was nearly the same, but the thickness got flat to about 2 to 3μm. This result is probably due to drying of mounting medium. Lymphocytes in air-dried Giemsa slide and improperly dried cells in Papanicolaou slides were highly flattened.

Small-sized cells like myoepithelial cells of breast duct, surface squamous epithelial cells, and shrunk cells in urine showed around 2μm thickness. If these cell mass is cut into 2μm layers, some of the nuclei may not come into focus on the virtual slide. Less than 2μm layer thickness is required for focus simulation. Thinner layer results fine pictures, but the thinner the layer set, the more the total number of layers increase. This causes longer scan time, more data size, and slower response to observe.

Thinking about total thickness to scan, number of stacked nuclei and the nuclear size should be considered. Papillary cell mass of urothelial carcinoma, aspiration cytology specimen of breast or thyroid, and uterine cervical smear showed different optimal thickness. Using our virtual slide, nuclei stacked more than 5 to 6 layers was difficult to distinguish. Stacked 5 nuclei of 2μm thickness each results in 10μm to scan. But usually carcinoma cells have big nuclei, as big as 50μm diameter and 10μm thickness. If these big cells stack together, we need to scan to 50μm or more. Usually, slides with more than 5 stacked cancer cell nuclei are exceptional and focal.

The “focus fusion” seems to contain all the cells in one plane, and easy for screening. But when trying to obtain detailed pictures like existence of myoepithelial cells, very important benign sign of breast, or to clarify the 3-dimensional tissue structure, multilayer image was better.

The efficiency of multilayer focus simulation depends on how easy we can change focus. The method to change focus differs from company to company[8]. Too many layer causes slow response. From these findings, we conclude 10 to 15 layers with 1.5μm thick each provide sufficient information in most specimens. In case of thick slide, 30μm thick scan may require.

## Conclusions

Cells in the cytology slides were smaller than the description in textbook. Layers of 1.5μm thickness each with total 10 to 15 layers resulting 15 to 20μm scan is suitable for most cytology slides.

## Competing interests

The authors declare that they have no competing interests.
